# An interdisciplinary and catchment approach to enhancing urban flood resilience: a Melbourne case

**DOI:** 10.1098/rsta.2019.0201

**Published:** 2020-02-17

**Authors:** B. C. Rogers, N. Bertram, B. Gersonius, A. Gunn, R. Löwe, C. Murphy, R. Pasman, M. Radhakrishnan, C. Urich, T. H. F. Wong, K. Arnbjerg-Nielsen

**Affiliations:** 1 Cooperative Research Centre for Water Sensitive Cities, Melbourne, Victoria, Australia; 2 School of Social Sciences, Monash University, Melbourne, Victoria, Australia; 3 Department of Civil Engineering, Monash University, Melbourne, Victoria, Australia; 4 Monash Art Design and Architecture (MADA), Monash University, Melbourne, Victoria, Australia; 5 UNESCO-IHE Institute for Water Education, Delft, The Netherlands; 6 Department of Environmental Engineering, Technical University of Denmark (DTU), Kgs. Lyngby, Denmark

**Keywords:** climate change adaptation, community visioning, flood resilience, interdisciplinary research, strategy testing, urban intensification

## Abstract

This paper presents a novel interdisciplinary and catchment-based approach for exploring urban flood resilience. Our research identified and developed a diverse set of adaptation measures for Elwood, a suburb in Melbourne, Australia, that is vulnerable to pluvial and coastal flooding. We drew on methods from social science, urban design and environmental engineering to gain integrated insights into the opportunities for Elwood to increase its flood resilience and urban liveability. Results showed that an appropriate balance of social, infrastructural and urban design responses would be required to retreat from, accommodate and protect against flood risk. These would also deliver broader benefits such as securing water supplies through harvested stormwater and mitigating extreme heat through greener landscapes. Our interdisciplinary approach demonstrated the value of (i) engaging with the community to understand their concerns, aspirations and adaptation ideas, (ii) exploring design measures that densify and use urban forms in ways that implement adaptation measures while responding to local context, (iii) adopting modelling techniques to test the performance, robustness and economic viability of possible adaptation solutions, and (iv) innovating governance arrangements and principles needed to improve flood resilience in the Elster Creek catchment. Our research also provided valuable insight on how to operationalize interdisciplinary work in practice, highlighting the importance of sharing an impact agenda, taking a place-based approach, developing a common conceptual framework, and fostering a constructive team culture.

This article is part of the theme issue ‘Urban flood resilience’.

## 1. Introduction

As city stakeholders grapple with the critical and complex challenges of population growth, urban densification and a changing climate, aspirations for urban liveability and resilience are becoming explicit in national and local policy, as well as international initiatives such as the Economist Global Liveability Ranking [1] and the Rockefeller Foundation's 100 Resilient Cities programme [2]. Flooding is a major threat to these aspirations [3]. More frequent and more intense storm events, combined with increasingly dense urban environments, means that communities are facing higher levels of flood risk, as well as other liveability threats such as reduced green space and deteriorating environmental health [4,5]. In the face of these challenges, the traditional approach to flood mitigation, which designs infrastructure based on frequency and magnitude of (models of) historical data, is becoming regarded by scholars (e.g. [6–9]) as less suitable; unable to deal with significant uncertainties in climate, urban development and society; and at risk of locking cities in to large-scale infrastructure investments that may not perform satisfactorily over the long term.

Methods that lead to more flexible outcomes are instead being advocated (e.g. [10,11]) and a new paradigm for land use planning, urban design and an integrated approach to water service delivery is emerging. This new paradigm recognizes that the flood risk profile of a city is less quantifiable under conditions of changing rainfall patterns and sea-level rise, and also that predictions of measures of risk can have unintended consequences such as creating a sense of complacency that can cause communities to be less prepared [12]. It also acknowledges the important role of public space, the built form and nature-based solutions in improving both a city's flood resilience and its liveability. For example, Rotterdam and Copenhagen have redesigned public spaces to cope with floods [6]: Rotterdam City Council has installed water squares that are used by the public for sports during dry periods but function as water detention tanks when it rains [13] and Copenhagen has redesigned roads and green infrastructure to allow pavements to be flooded up to 10 cm in a 100-year cloudburst event to cope with the impacts of flooding.

Embedding this paradigm in practice requires cities to adapt through the implementation of innovative structural and non-structural measures [14–16]. Contemporary approaches incorporate a sophisticated structural mix of green and grey initiatives (e.g. pumps, flood walls, buildings, nature-based technologies, public open spaces) that are embedded through design processes into the urban form, supported by non-structural initiatives that focus on society and institutions (e.g. policy, regulation, information, behaviour, governance processes).

Against this background, we propose that an integrated flood resilience strategy not only improves a city's capacity to cope with floods of various magnitudes, but also supports a city's liveability objectives by delivering broader social, environmental and economic benefits (such as securing water supplies through harvested stormwater and mitigating extreme heat through greener streets and landscapes). In this process, an integrated and coherent suite of structural and non-structural adaptation measures optimize the use of land and resources. This paper demonstrates and reflects on the application of an interdisciplinary and catchment-based approach for developing such a strategy. It integrates results from social science, urban design and environmental engineering methods to develop interdisciplinary insights into the opportunities for increasing a local area's flood resilience and urban liveability through a range of adaptation measures, as well as guidance on how to operationalize interdisciplinary research effectively. This collaborative project was conducted through a case study of flooding in Elwood, a coastal suburb of Melbourne, Australia.

## 2. Urban flood resilience framework

Understanding flood risk and the range of available management responses that may be suitable for a particular context is clearly necessary for developing an effective suite of adaptation measures. Many frameworks have been developed worldwide that can provide meaningful insight to support such analysis and inform flood risk management policy, each with a particular purpose and focus (see [17–20] for recent examples). Our study, focused on measures to increase flood resilience while delivering broader liveability benefits, required a broad organizing framework that could provide a familiar and common base for identifying local-scale social, policy, technical and design interventions. To this end, we adopted a three-tier framework for climate adaptation proposed by Gilbert & Vellinga's [21] in work for the Intergovernmental Panel on Climate Change (IPCC), and further developed by Klein *et al*. [22]. It sets out three types of adaptation options: manage the retreat from vulnerable areas and resettle inhabitants; accommodate continued occupancy and use of vulnerable areas; protect vulnerable areas with defence approaches. While the original framework focused on infrastructural responses to coastal flooding, here we adapt and expand it for use in developing and prioritizing policy, infrastructure and urban design adaptation measures that address social and financial vulnerability and resilience to fluvial, pluvial and coastal flooding:

— **Retreat:** Reassign land use for purposes that will not be adversely affected by flooding (e.g. through controlling housing density, creating ecological landscapes and parks, and converting land for food production) [23,24]. The Room for the River program in The Netherlands [25] exemplifies this tier. Areas frequently affected by flooding would be well suited to the retreat strategy.
— **Accommodate:** Accommodate flooding by adapting the built form through urban planning and design innovation, as well as public learning and awareness (e.g. changes in housing typology, changes in the use of upper and lower storeys of buildings, installation of waterproofing fittings on buildings, integrating open spaces and green corridors into the drainage network to convey stormwater flows, contingency planning, societal awareness and capacity building) [26,27]. The Dutch innovation of floating houses (e.g. IJburg in Amsterdam) illustrates this type of response. Areas affected by moderate flood events would be well suited to accommodate the strategy.
— **Protect:** Defend against flooding through engineered infrastructure (e.g. pumps, flood gates), ecosystem-based solutions (e.g. wetlands, mangroves) and emergency management systems (e.g. enhanced warning systems, emergency response plans) [22,28]. The Dutch system of polders and dykes are examples of responses that aim to defend. Areas affected by infrequent but major flood events would be well suited to the protect strategy.

Guided by this framework, we characterize flood resilience as having sufficient capacity in a catchment to manage the impacts of flood events within a community's level of tolerance, and reducing exposure of people and property to flood risk in the longer term. This is achieved through selected measures for adaptation that respond to local socio-technical conditions and achieve an appropriate balance of investment in social and infrastructural dimensions [29,30]. The suitability of a measure may depend on a location's relative vulnerability to fluvial, pluvial or coastal flooding, or a combination of all three. Measures that focus on housing design can reduce flood hazards through raising floor levels, introducing waterproof doors and windows, and ensuring sleeping quarters are well above predicted flood levels; however, these can only be effective with accompanying increased awareness and flood preparedness of the people living within. Measures that establish designated flow paths and flood detention can be particularly effective in managing pluvial flooding, but need to be integrated in the urban designs of local areas. An over-reliance on infrastructure may have major cost implications which would need to be evaluated within the context of a community's tolerance levels for flood impacts. Measures may provide additional secondary benefits that should be considered, such as improved liveability, stormwater cleansing and ecological biodiversity in the urban environment. Ultimately, a comprehensive flood resilience strategy will need to incorporate a suite of measures that address objectives associated with (i) preparedness to mitigate flood hazards; (ii) infrastructure defences and community responses during the occurrence of a flood event; and (iii) recovery following a flood event.

The complexity and interconnectedness of urban flood risk management issues must be reflected in decision-making [31]. This includes the need for interventions to deliver multiple benefits to meet broader liveability objectives in addition to flood risk management [32]; intertemporal complexity relating to uncertainties associated with climate change or social–ecological systems [33,34]; and the need to engage with different forms of knowledge and expertise to address gaps in scientific understanding [35,36]. Dealing with this complexity and uncertainty requires flood risk managers to engage with actors of diverse knowledge and power in processes of mutual interaction, reflexivity, coevolution, co-production, experimentation and learning [37–41].

The demands associated with the complexity described above require better coordination of insight and perspective across organizational and disciplinary silos, as well as close integration of research and policy development processes [42]. More specifically, an urban flood resilience strategy guided by the framework developed in this section should be underpinned by insights from urban planning, infrastructure design, community participation and governance. Development of such a strategy necessarily involves multiple disciplines to support the identification and delivery of a suite of technical, design and policy measures that fit well with a catchment's cultural, geographical and ecological context, and that are supported by corresponding governance arrangements.

## 3. Methods

### (a) Interdisciplinary research platform

The Cooperative Research Centre for Water Sensitive Cities [43] is an Australian Government, university and industry partnership between over 85 organizations, including state agencies, local municipalities, water utilities and private companies. Its aim is to undertake and support the adoption of interdisciplinary research to transform water management to deliver multiple liveability, sustainability, resilience and productivity benefits (e.g. water supply security, resource efficiency, greener and cooler landscapes, and healthy waterways), represented by the concept of the water sensitive city. The CRCWSC provides a significant platform for enabling and promoting efforts to consider topics like flood resilience through an integrated lens.

**Figure 1.**
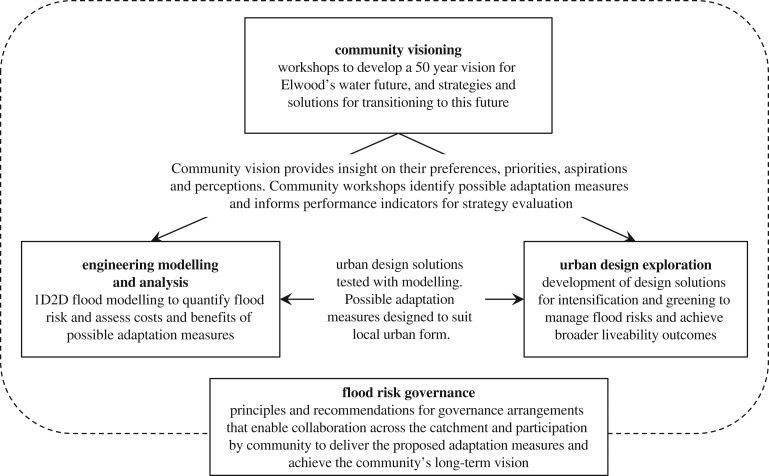
Contributions of individual CRCWSC projects for the integrated Elwood case study.

A number of individual projects within the CRCWSC selected Elwood as a case study, each bringing their particular disciplinary view and specific objectives to develop an integrated understanding of the issues and opportunities in relation to flood risk management for the suburb (figure 1).

### (b) Case selection

**Figure 2.**
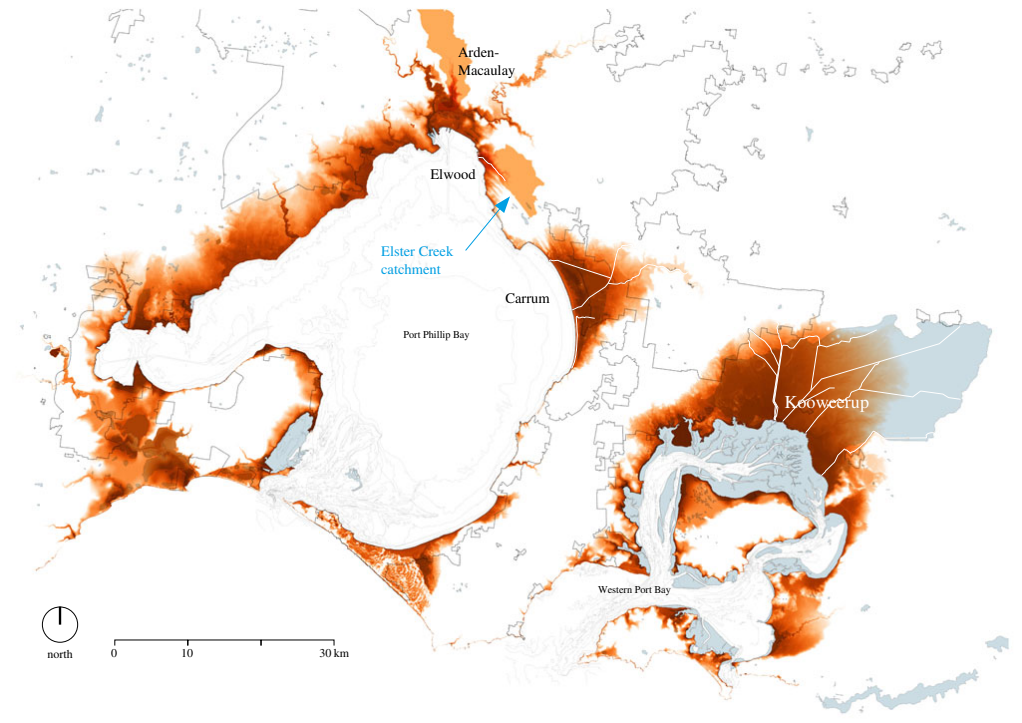
Elwood is one of Melbourne's low-lying suburbs around Port Phillip Bay and part of the Elster Creek catchment (image source: MADA). (Online version in colour.)

Elwood, a suburb approximately 10 km southeast of Melbourne's Central Business District, is facing a range of long-term planning and water management challenges, providing a fertile context for analysis. Elwood is home to 15 543 residents [44] who live in a mix of apartments, townhouses and detached dwellings, and enjoy urban services and aesthetics corresponding to the suburb's high median personal income [45], as well as a strong sense of community [46]. It is a coastal suburb and has a significant area of drained swampland, lying at the bottom of the 40 km^2^ urban catchment of Elster Creek (figure 2). Elster Creek is now a highly modified watercourse, only surfacing in its final 4 km in concrete-lined channels. These factors contribute to the suburb facing flood risk, which is predicted to significantly increase with climate change due to more frequent and more intense rainfall events and rising sea level. Long-term climate models have also projected hotter and drier conditions, presenting regional water scarcity and urban heat island challenges [47]. Pollution in the Elster Creek is also a regular issue for residents [46].

These issues have prompted reconsideration of water management at the local and metropolitan planning levels. For example, planning controls across the local municipality were recently reviewed to minimize the impact of 100-year flood events on new development. The local community has experienced a number of flood events in recent years, and are also aware of broader sustainability and liveability issues. They are active and vocal citizens, passionately expressing concerns and priorities in various consultation processes with the City of Port Phillip (Elwood's municipal Council). However, flooding issues are obviously influenced by decisions upstream of Elwood, which includes three additional local governmental areas (City of Glen Eira, City of Bayside and City of Kingston), as well as other public agencies that have responsibilities for flood management in the catchment (elaborated in §4c).

### (c) Community visioning

Community concerns, priorities, aspirations and ideas for flood resilience measures were sought through a series of three participatory workshops with local citizens of Elwood (*N *= 24, 17 and 18 for the three workshops). Each workshop was 3 hours long, taking place in July, August and September 2015. The principles underpinning the workshop methodology are outlined below; more detailed information and analysis about the process and outcomes is published elsewhere [46].

The workshops were based on a transition scenario methodology (e.g. [48–50]), which aims to examine significant long-term sustainability challenges and future aspirations, drawing on principles of envisioning and backcasting. The approach aims to bring stakeholders together to generate a shared understanding and motivation for enabling transformative change, develop a common vision to guide action and identify pathways for change that offer a roadmap for navigating change processes. Participation by local stakeholders is particularly important to ensure local knowledge informs effective identification of transition pathways and to translate universal principles of sustainability in urban environments to a specific local context. It can also stimulate learning among participants, both in terms of awareness of the issues being explored and in developing new perceptions to drive an ongoing change agenda [51]. Following participation in such processes, individuals may identify opportunities for practice change or innovation that can contribute to bringing about the future vision or opportunities for joint action [50].

For this research, the focus was on developing a community-led vision and strategy for achieving a water sensitive Elwood across a 50-year time horizon to 2065. Community discussions were supported by the provision of stimulus material by the research team's facilitators. Subsequent analysis of the vision and strategy by the integrated research team derived a suite of flood resilience measures to adapt the biophysical environment, which then informed the urban design and modelling research. The community also proposed ideas for improving flood risk governance in the catchment. The project timelines did not provide the community participants with opportunity to refine their ideas following the urban design and engineering modelling; this would be a valuable further methodological step in future processes.

### (d) Urban design exploration

The community vision and adaptation measures informed the development of novel design solutions for urban densification that also addressed flood risk. This involved a design-led methodology [52–54] that investigated the underlying nature of water in urban contexts by studying its history, modification, management and control as an entry point for constructively and tangibly engaging with complex issues of climate change, resilience and the interrelationships between natural, infrastructural and human elements. By elevating and prioritizing water as the driver of urban form in Elwood, the design approach explored how to unlock new thinking about water systems in place. This was partly informed by case study research from other places, such as small/medium scale projects that keep water on site (e.g. Stawell Steps [55], Rotterdam Water Squares [13]) and design speculations developed through workshops, competitions and exhibitions, which later might be adopted as policy or as initiators for projects (e.g. Rising Currents [56], Design in the Terrain of Water [57]).

The design research team engaged in an iterative process of immersion through field experience, analysis through drawing, collaboration through engagement with the other disciplines, speculation through design studios, and debate through discussion, exhibition and design charrette [52]. This led to a design synthesis of the community ideas and subsequent testing with engineering models, as well as a design studio (Swamp City) facilitated with Masters of Architecture students that developed and focused a critical mass of design ideas and production to directly inform the research. Outcomes were developed into a linked set of propositional and, in some instances, provocative adaptation strategies that were exhibited in a local gallery to key stakeholders and the general public. The exhibition aimed to raise awareness of potential solutions and processes through visualizing and analysing cumulative change scenarios, which ranged in scale from catchment-wide thinking to key local Elwood sites identified for immediate action.

### (e) Engineering modelling and analysis

A computational decision-support tool was developed and applied to analyse the interactions between urban forms, infrastructures and flood risk in the Elster Creek catchment. This enabled the flood mitigation potential and associated costs and benefits of infrastructure solutions and innovative urban designs to be tested under different scenarios of urban development and climate. The testing was enabled by recent developments in scenario modelling to make faster and more computationally efficient models with realistic representations of future scenarios, as demonstrated in [58–60]. The modelling was supported by DAnCE4Water's urban development algorithms [61] coupled with the MIKE FLOOD software that assesses flood risk in space and time [58,62–64].

Testing mitigation measures for different potential futures allowed solutions that will be robust under many different scenarios to be identified. The detailed modelling set-up helped consideration of a wider range of adaptation options and at different spatial scales, for example through spatially explicit and integrated modelling of options. The modelling approach thus enabled testing of the efficiency of measures ranging from lot-scale rainwater harvesting tanks to catchment-scale retention basins, as well as other installations, such as constructed wetlands, that have both water management and broader liveability and sustainability functions that the community articulated as important through the participatory planning process. The modelling also enabled exploration and quantification of benefit transfers between different locations. In other words, the spatial distribution of benefits and costs and the extent of impacts for different parts of the catchment across physical or administrative boundaries could be analysed. While Elwood is not home to particularly vulnerable communities, this type of spatial analysis could allow for areas to be targeted for maximum benefit, and provide important insight for policy-making and planning to ensure the wellbeing of all communities in a catchment (see [65] for a brief discussion on ethical issues in relation to implementing flood risk measures).

### (f) Flood risk governance

Analysis of the current governance arrangements for flood management in the Elster Creek catchment was conducted through a review of secondary documentation, including policy materials, organizational reports and scientific articles on water governance in Melbourne (e.g. [66]). Recent developments in the Elster Creek flood risk governance following this integrated research were directly observed through the research team's participation in Working Group meetings among industry stakeholders, which aimed to respond to key recommendations derived through the research. A review of scientific literature on water governance, adaptive governance and community resilience more specifically identified key principles and recommendations emerging from the scholarship for improved governance approaches in the context of complex, uncertain and changing environmental conditions that characterizes the Elster Creek catchment.

## 4. Results

We now present a synthesis of the findings from the interdisciplinary research activities described in §3, analysed through the three-tier framework for guiding flood resilience adaptations. References to publications with more detailed results from the individual constituent projects are indicated; this paper focuses on highlighting how the integrated research individually and collectively informed the development of a wide range of technical, urban design and social measures to increase flood resilience.

### (a) Elwood's water future

**Figure 3.**
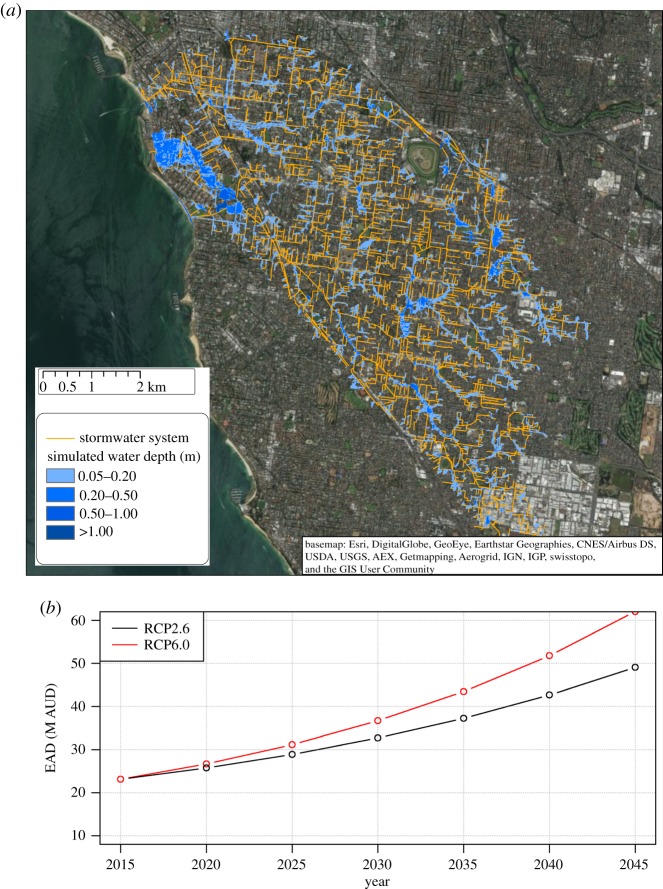
Modelled future scenarios for Elwood. RCP, representative concentration pathway. Image for (*a*) reproduced from Radhakrishnan *et al*. [60] with permission from journal. (*a*) One in 100-year pluvial flood event in a business-as-usual scenario in 2090. (*b*) Change of expected annual damage under different climate scenarios (RCP2.6 and RCP6.0). (Online version in colour.)

Flood risk modelling showed that both pluvial and sea surge hazards are substantial in the Elster Creek catchment. In a business-as-usual scenario in 2090, the downstream part of the catchment is particularly vulnerable to flooding caused by storm surges and there are significant vulnerabilities to a 1-in-100 year pluvial flood event in the catchment's upstream areas (figure 3*a*). The analysis showed that the expected annual damage in the catchment will increase with climate change (figure 3*b*), largely driven by sea-level rise. Uncontrolled urban growth will further increase flood risk in the Elster Creek catchment, due to the increased impervious area and development occurring in low-lying areas that experience frequent flooding.

**Figure 4.**
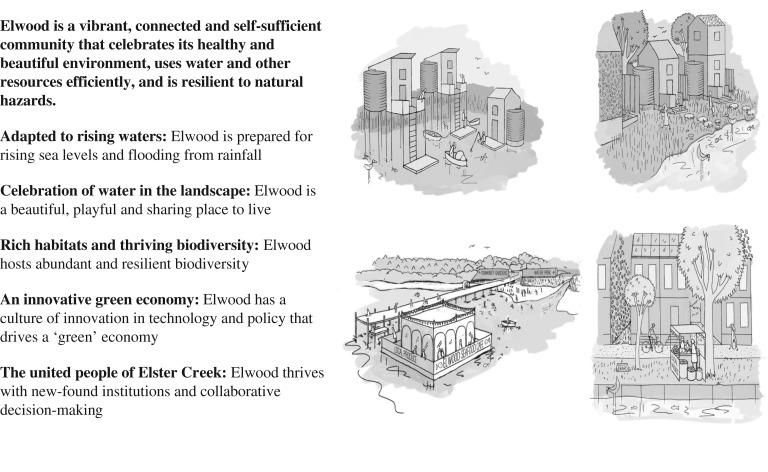
Community participants' water-sensitive vision for Elwood in 2065 (Rogers & Gunn [46]; images: Wendy Tyrer).

While these modelling results were not provided to the community participants, the workshop process revealed their strong concern about the future of Elwood with respect to flooding, and flood resilience was central to the participants' discussions. However, their long-term aspirational vision for Elwood's water and urban environment also encompassed a broader range of liveability, sustainability and resilience values, as represented in their overarching vision statement and associated synergistic themes [46] (figure 4).

Each vision theme comprised a wide range of ideas for adaptation measures across social, technical and ecological domains that elaborated the key concepts underpinning the vision of Elwood's water future. ‘Adapted to rising waters’ was based on a recognition of greatly increased incidence of flooding and included urban landscape adaptations such as water storage within wide streets, laneways with permeable paving and bioretention systems and an artificial reef to mitigate storm surges. ‘Celebration of water in the landscape’ involved ideas for introducing local food production through community gardening and aquaculture and using schools in the catchment as demonstration sites. ‘Rich habitats and thriving biodiversity’ involved drawing on Australian Indigenous people's knowledge about natural resource management, restoring wetlands and pursuing integrated urban planning and management around the canal. ‘An innovative green economy’ included water sensitive and low carbon designs such as green roofs and walls in public and private realms, implementing water sensitive urban renewal projects, harvesting stormwater for reuse and undertaking integrated planning of urban services, including building, transport, energy and water. ‘The united people of Elster Creek’ involved uniform catchment regulation, collaborative decision-making, fostering cross-jurisdictional and multi-institutional collaboration, fostering co-design opportunities, and sharing information and responsibility for managing flood risk and mitigating action across the catchment.

### (b) Developing adaptation measures

Significant adaptations will be needed to mitigate and manage the flood impacts anticipated by the modelling. However, the community's vision for Elwood's water sensitive future highlights that mitigation of flood impacts is not their only priority: any adaptation measure must preserve and enhance the range of broader liveability and sustainability values. In the Australian context, ensuring water supply security and mitigating extreme urban heat are particularly pressing issues that could be addressed through innovative flood management strategies. Possible adaptation measures identified by community participants addressed challenges such as the imperviousness of the catchment, the poor waterway health of Elster Creek and the declining amenity of the urban environment. Their ideas for biophysical adaptations cover four key themes that would need to be collectively pursued: managing flood paths to maximize community benefit, greening streets and lanes, naturalizing the canal, and protecting and embracing coastal life.

Developing these potential biophysical adaptation measures into implementable options for the Elster Creek catchment requires evaluation of their feasibility and likely efficiency (defined as benefit-cost ratios in table 1), along with explicit consideration of the form and function of the local urban environments and other social, ecological and technical conditions. The urban design and modelling processes helped with this, exploring possible realizations of catchment- and local-scale adaptation measures that would not only achieve flood mitigation measures but also respond to the community's broader values and aspirations. The efficiency of each measure in reducing flood risk was analysed under both the IPCC Representative Concentration Pathway (RCP) 2.6 and RCP 6.0 scenarios to obtain (some) insight into the temporal dimension of efficiency. Analysing the spatial sensitivity of the adaptation measures is beyond the scope of this paper, but details about the spatial variations can be found in [59].

**Table 1.** Further development of measures for enhancing Elwood's flood resilience. Ca, catchment; Sw, swamp; Co, coastal.

				community vision themes^b^						net BCR in RCP^c^	
potential adaptation measures		suitable landscape types	event type managed^a^	a	b	c	d	e	investment (M AUD)	2.6	6.0
*retreat* making room for Elster Creek flow and sea-level rise, for example:	a. Creating ecological landscapes	Ca, Sw, Co	large	X	X	X					
	b. Detaining floods in upstream open space	Ca	medium	X	X		X		13.4	4.4	5.1
	c. Designating flood ways	Sw, Co	large	X	X	X					
	d. Purchasing high flood risk properties and converting to non-residential uses	Sw, Co	large	X			X				
*accommodate* design houses and urban spaces, as well as temporary measures, to protect homes from stormwater, for example:	e. Setting floor levels higher and making appropriate use of ground floor spaces	Sw	large	X					236.9	1.4	1.7
	f. Increasing permeability of surfaces	Ca, Sw, Co	small	X	X	X					
	g. Encouraging installation of rainwater tanks on private property	Ca, Sw, Co	small–medium	X		X			166.0	1.8	2.0
	h. Using public spaces for retention	Ca, Sw, Co	small–medium	X	X		X		1.5	24.7	28.8
	i. Building social capital for planning and preparedness	Ca, Sw, Co	large	X			X				
	j. Developing emergency responses	Sw, Co	large	X			X				
	k. Preparing for recovery after flood events	Sw, Co	large	X			X				
*protect* install major infrastructure to defend against flood events, for example:	l. Flood gates and pumps in combination with large-scale retention in the shore area and the catchment	Ca, Sw, Co	small–large	X	X				139.9	0.7	0.9
	m. Levees around key assets	Sw, Co	large	X							
	n. Large increase in pipe capacity and installation of diversion pipes	Sw, Co	small–large	X					996.0	−0.6	−0.6

^a^Indicative average recurrence interval (ARI) of event managed by adaptation measure: large, greater than 100 years; medium, 20–50 years; small, less than 10 years.

^b^a, adapted to rising waters; b, celebration of water in the landscape; c, rich habitats and thriving biodiversity; d, an innovative green economy; e, the united people of Elster Creek.

^c^Costs and benefit–cost ratios (BCR) that were calculated in the analysis for representative concentration pathways (RCP; or greenhouse gas concentration trajectories) 2.6 and 6.0 are shown.

**Figure 5.**
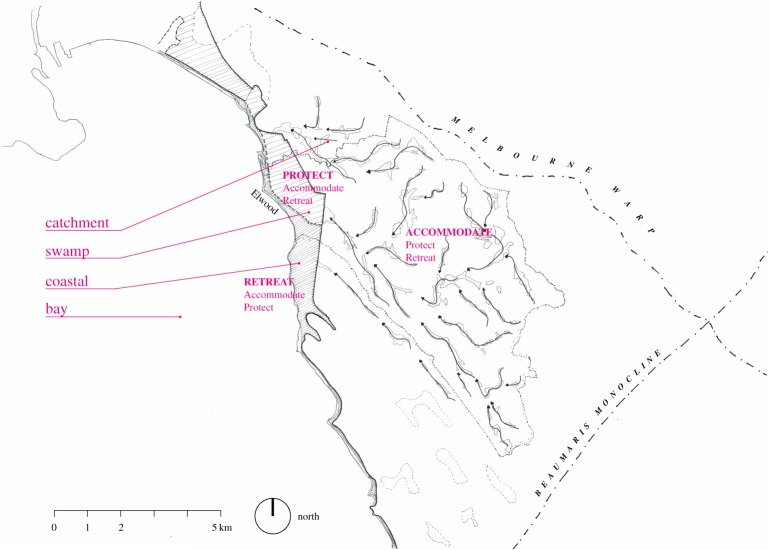
Landscape types and corresponding adaptation strategies (image source: MADA). (Online version in colour.)

**Figure 6.**
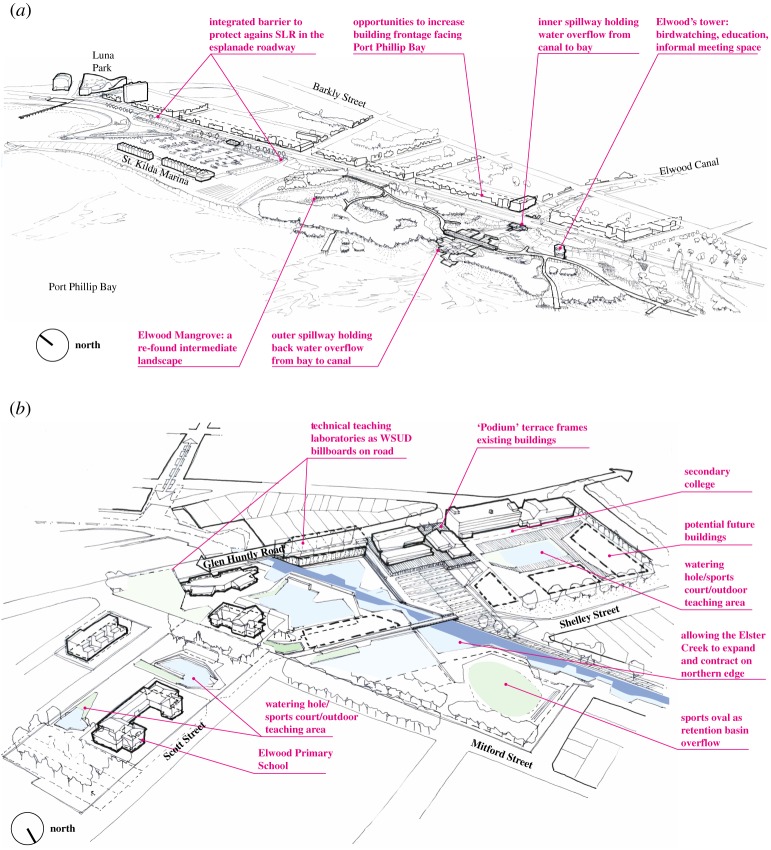
Adaptation measures to (*a*) protect Elwood's coastal zone and (*b*) retreat from and accommodate pluvial flooding in Elwood's swamp zone (image source: MADA). (Online version in colour.)

Design analysis across the Elster Creek catchment identified four landscape types: catchment, swamp, coastal and bay, each suitable for its own suite of potential adaptation measures (figure 5). For example, coastal areas may require protect measures such as implementing surge control structures to deal with rising sea levels, storm surge and the unpredictable and often heavy outflow at the mouth of Elster Creek predicted by the modelling (figure 6*a*). The community reimagined the coastal area as a recreational attraction and ecological asset, centred on a mangrove barrier that could prevent the intrusion of seawater up the canal and limit the outflow of polluted freshwater to protect the health of the marine environment. This idea was developed further by the engineering and design researchers, proposing a mangrove landscape (common on the coastline of Victoria) on the foreshore and two spillways that would control the inflow and outflow of Elster Creek. The mangroves provide amenity and increased biodiversity, as well as storage capacity for excess water during storm events.

By contrast, mitigation of flood risk in downstream (former) swamp areas may require retreat measures such as the buyback of properties in flood zones or the redesign of vulnerable sites to accommodate pluvial flooding (figure 6*b*). The community proposed that the grounds of two schools in the swamp landscape could be redesigned to incorporate a series of water squares linked through pipes or open gutters, which allow water to flow throughout the site when it rains and echoes a chain of billabongs (stillwater ponds) in the natural environment. These ideas were further developed by the engineering and design researchers, proposing that stormwater runoff from school building roofs and nearby streets be captured and collected in these squares, and treated through biofiltration technologies that border the squares before it is released back into the system. These water spaces provide benefits in both wet and dry periods, through educational, recreational and sporting uses.

Table 1 presents the set of potential adaptation measures that emerged through the research and were considered for further development for the Elwood case study to explore how they could be feasibly implemented to address Elwood's flooding challenges. These measures draw on both the ideas proposed by the community and analysis of the modelling results. They are organized according to the three-tier urban flood resilience framework developed in §2, specifying whether they take a retreat, accommodate or protect approach and the landscape type and event magnitude they are most suitable for. They also have additional benefits, as indicated by the corresponding future vision themes, thus potentially appealing to different stakeholders.

**Figure 7.**
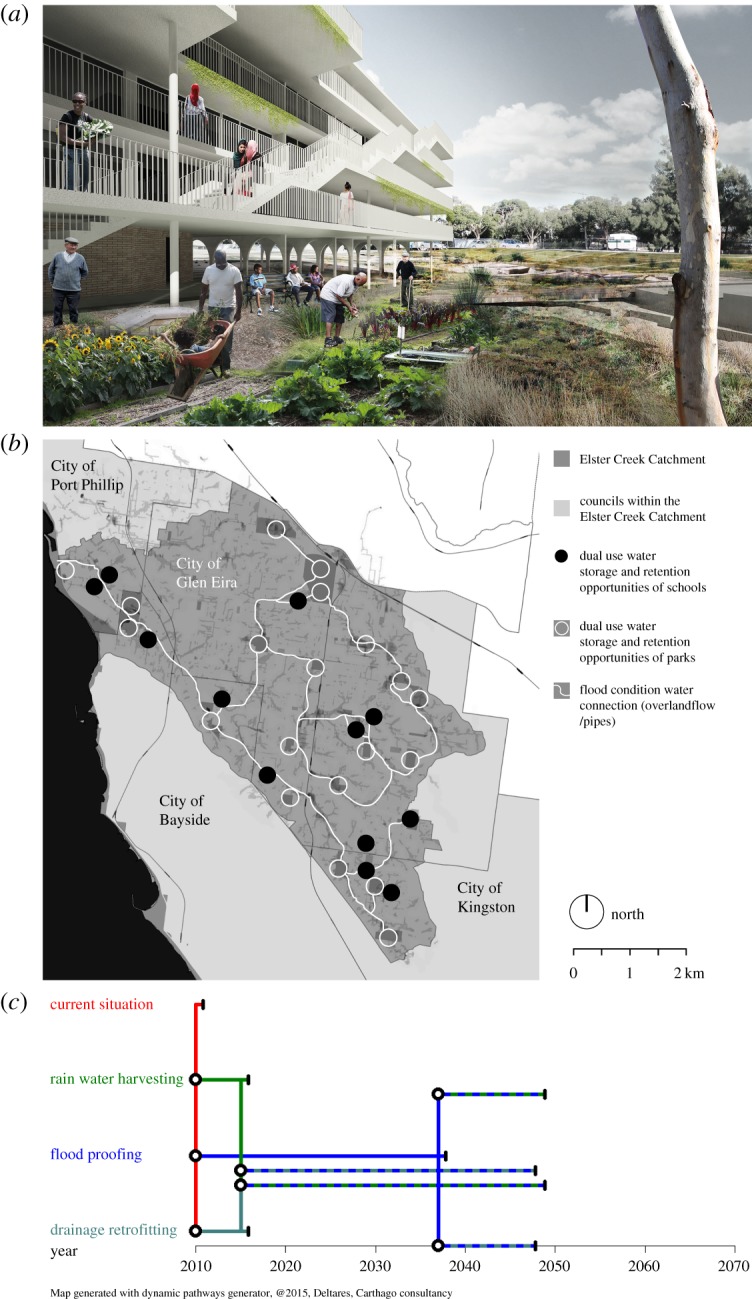
Examples of how potential adaptation measures were further investigated. (*a*) Using and adapting the existing building stock to accommodate flood events (image source: MADA). (*b*) Catchment-wide network of public open spaces to increase flood retention capacity and mediate storm surge impacts (image source: MADA). (*c*) Implementation pathways for possible adaptation measures under climate scenario RCP 2.6. Black vertical lines indicate the median year for a tipping point, with the grey bar representing its range. (Online version in colour.)

Figure 7*a* illustrates measures that use and adapt the existing building stock (measure e in table 1), 75% of which in Elwood comprises walk-up flats or apartments. This building type and scale has substantial inertia due to strata-titling, but also offers unique possibilities for adaptation. For example, concrete structures raised on columns allow incentives to be put in place to remove ground-level dwellings and replace them with new floors of accommodation on top of the existing structure. This requires planning envelope height limits to be raised in order to incentivize more openness and porosity to water flow at ground level. Additional incentives for retrofitting elevators for accessibility, or implementing community re-vegetation or flood refuge spaces could also be given through devices such as rate holidays for participating body corporates.

Figure 7*b* examines connecting up a catchment-wide network of public open spaces to increase flood retention capacity and mediate the impact of storm surge on the inner wetland environment (measures b and h in table 1). Existing parks, local school grounds, sports ovals and the foreshore reserve are proposed with multi-purpose criteria as dual-use public infrastructure that changes purpose between wet and dry modes.

Figure 7*c* illustrates the importance of understanding the sequential investment that would be needed to most efficiently implement different adaptation measures to inform planning and decision-making, presenting an example result from the many adaptation pathway analyses undertaken. This type of analysis provides insight into how long a given measure can provide adequate flood protection. For the Elster Creek catchment analysis, adequate flood protection was defined as annual flood damages below 0.5% of the net revenue generated in the catchment annually and a tipping point occurs when damages exceed this level (see [56] for relevant assumptions about climate change and urban form). In this example, the initial situation (2010) is already at a tipping point, so immediate action is needed, and three potential adaptation measures are considered: rainwater harvesting (measure g in table 1), flood proofing of households through building changes (measure e in table 1) and retrofitting drainage to increase capacity (measure n in table 1). All three measures would help mitigate flood risk in the short term (to 2015), but flood proofing of households is needed to yield sufficient protection when the planning horizon extends beyond this. The adaptation pathways show that sufficient protection would be provided if initial investment in rainwater harvesting was followed by flood proofing measures in 2015. Alternatively, initial investment in flood proofing could be followed by rainwater harvesting or drainage retrofitting in 2037.

### (c) Governance implications

Implementing novel retreat, accommodate and protect measures will have implications for the governance arrangements associated with the management of water and the urban form. The current arrangements for governing drainage, flood events and associated impacts in Elwood and the Elster Creek catchment are complex, with responsibility shared across the Victorian Government, two state-owned water corporations, four municipal Councils and individual property owners. While these arrangements have performed well in many respects, the community's experience of persistent flooding impacts highlights the need for improvement.

The importance of transboundary cooperation to manage flood risk across political boundaries is well recognized in the scholarship (e.g. [21]) and was understood by the Elwood community participants. They identified that a lack of coordination between local agencies meant that a more holistic and integrated approach to flood risk management was difficult to achieve. To address this problem, they envisioned a catchment-wide approach to flood risk management and proposed the hypothetical idea of the ‘City of Elster Creek’, whereby the Elster Creek system would be the defining element in determining where administrative boundaries were drawn. While it may not be feasible to establish catchment-based municipal boundaries, enhanced coordination and collaboration across agencies with flood-related responsibilities in the catchment should be.

Indeed, approximately six months after the findings of the community envisioning process were reported, a Working Group of the key agencies operating in the Elster Creek catchment was established, facilitated initially by the CRCWSC. Its objectives were to reflect on the insights from this research and explore the potential of a collaborative catchment-wide approach to develop consistent policy across the catchment, share information and explore technical solutions with potential catchment-scale benefits. The Working Group then arranged a number of interagency forums with Mayors and CEOs to engage political and executive decision-makers in discussion about working together to achieve catchment-level outcomes. A Memorandum of Understanding was signed by all parties and an ongoing governance arrangement, the Elster Creek Catchment Forum, was established to drive a strategic Action Plan, incorporating short-term actions, resourcing requirements and timelines for deliverables. Key activities include developing a community engagement approach, compiling data and doing comprehensive mapping, and assessing data to develop options and identify best-fit solutions.

This governance outcome is promising for stakeholders to deliver on the potential of the integrated solutions explored through this research. Its success may depend on how well key principles espoused by recent scholarship on water governance are adopted. Effective flood risk governance would require ‘enabling and supporting adaptive institutions that are able to cope with complexity and uncertainty in the face of new challenges and possible surprises' ([34], (p. 80)). For the newly established Elster Creek Catchment Forum, this means operating within a long-term planning horizon and adopting a responsive, anticipatory approach to flood policy and mitigation options [23,34]. Increasing adaptive capacity is critical and scholars recommend a range of approaches for achieving it in practice.

— Maintain an array of options to adopt in response to plausible scenarios that consider biophysical and social adaptation responses, impacts and uncertainties [24,67].
— Facilitate more symmetrical forms of engagement that do not automatically privilege some ideas, actors or practices [68,69].
— Foster the responsiveness of institutions [33] and establish regulatory flexibility that provides a response framework that can be applied in diverse situations [40].
— Establish governance arrangements that support multi-level decision-making and a balance between bottom-up and top-down processes [40,70].
— Seek clarity about the groups affected by floods and ‘who has the responsibility, capacities, access to resources and information’ to manage the problem [34, p. 70] and involve them in planning and decision-making to respond to community needs over a top-down approach [33,71].

Finally, our engagement with the Elwood community revealed they see immense benefit in being considered partners in flood risk governance for the Elster Creek catchment, confirming the benefits identified in the literature. This includes using local knowledge for planning and flood recovery [36,72], increasing community agency to take flood resilience action [73,74], gaining insight into the needs of at-risk communities [74], improving implementation and maintenance of local adaptation technologies [22] and increasing public support and legitimacy for changes along with effective local action [75]. The community participants were capable of grappling with the complexities and uncertainties inherent in the discussions and valued the opportunity to be engaged in discussions about planning for the long-term future. They helped to reframe flooding problems in ways that were most relevant for the local community, came up with many creative and strategic ideas and solutions for technical, design and policy adaptation measures, and recognized their role in contributing to flood resilience through measures that can be implemented in their own private realm.

## 5. Discussion

### (a) The value of interdisciplinarity for flood resilience

Examining the results from the three integrated areas of research activity highlights that an interdisciplinary approach has enabled deeper and broader exploration of the opportunities for increasing flood resilience in Elwood than any individual approach could achieve in isolation.

A range of insights from the community visioning process could contribute to enhancing Elwood's flood resilience. These include innovative local adaptation measures, understanding of what the community would deem acceptable in terms of flood resilient strategies and solutions, and articulation of broader liveability and sustainability values that need to be considered while pursuing increased flood resilience.

The urban design research interpreted and integrated ideas for adaptation into feasible designs that would fit with specific landscapes and deliver multiple benefits to address broad liveability aspirations. These contributions were important for supporting the development and analysis of flood mitigation options, particularly in translating ideas across social, technical and ecological domains and visualizing landscape adaptations that would be a radical change for the community [52].

The modelling work supported analysis of possible biophysical adaptation measures through quantification of flood risk vulnerability of local areas, as well as the costs and benefits of different options, including both flood resilience benefits and co-benefits that support broader liveability and sustainability. This type of analysis was valuable for evaluating combinations of design adaptations from lot to catchment scale, informing the design of major flood defence infrastructure and supporting the development of emergency response plans.

The analysis of flood risk governance suggested arrangements for better implementation of a flood resilience agenda and the establishment of a network of local community and agency champions who are motivated to undertake action for improving Elwood's flood resilience. This analysis enhanced the feasibility of the adaptation measures and ensured the consistency of planning policy.

Table 2 synthesizes the key contributions from each disciplinary perspective in developing a wide range of technical, urban design and social measures to increase flood resilience, organized according to the three-tier flood resilience framework. It highlights the value of integrating the community visioning, urban design and engineering modelling approaches to develop complementary and synergistic insights that significantly enhanced the relevance, feasibility and acceptability of identified options. It also enabled a broad view that prioritized liveability and sustainability benefits in addition to the core objective of flood resilience.

**Table 2.** Disciplinary insights that contribute to increasing flood resilience for Elwood.

adaptation strategy	community visioning	urban design expression	engineering modelling and analysis	flood risk governance
*overarching*	understanding the capacities for community members to partner in flood risk governance and implement adaptation measures	interpreting ideas for adaptations into feasible urban designs developing design visualizations to support options analysis	developing flood risk visualizations to support options analysis quantifying the costs for biophysical adaptation options	ensuring consistency of planning policy across the catchment ensuring equity in the distribution of flood adaptation and mitigation costs, benefits and risks
*retreat:* use areas frequently affected by flooding for other purposes	understanding community values and priorities about the local area identifying local areas and streets suitable for designating as greenspace and floodways	developing site specific designs for ecological landscapes that provide room for floodwaters	identifying areas of high flood risk that could be converted to other land uses	integrating public participation in governance processes to increase understanding of risk and acceptance of retreat solutions, as well as to manage potential opposition establishing flood zone maps in relation to catchment boundaries rather than administrative boundaries
*accommodate:* adapt the built form to accommodate moderate storm events	understanding community tolerance for flood level and frequencies sourcing ideas for adaptations in the private and public realm developing strategies for engaging citizens in behaviour change and education campaigns	developing housing and urban form typologies that protect property from floodwater while delivering broader liveability outcomes	quantifying the flood risk vulnerability of local areas identifying combinations of design adaptations (from lot to catchment scale) that reduce flood risk vulnerability to an acceptable level highlighting and quantifying the potential multi-functionality of design adaptations	involving community in participatory forums to contribute to implementation, monitoring and evaluation of adaptation solutions
*protect:* implement major infrastructure and emergency response measures to defend against major floods	understanding broad community objectives, including liveability values and critical areas that require defence understanding the potential for community members to contribute to emergency response and recovery activities	integrating major flood mitigation infrastructure works into multi-functional landscapes	informing the design of major flood defence infrastructure adaptations, including type, size and location supporting the development of emergency response plans	overcoming local opposition to infrastructure proposals using deliberative decision-making processes

### (b) Insights for doing interdisciplinary work

By actively seeking input from disciplines outside the traditional siloed repertoire of engineers, such as urban design and social science, our understanding of the Elster Creek's systems, structures and processes was individually challenged and collectively expanded in a way that better equipped the team to engage with the complex, real-world problem of Elwood's flood resilience. The process of interdisciplinary collaboration, however, needed careful navigation by the research team to ensure opportunities and challenges were well managed. Brown *et al*. [76] provide guidance on how interdisciplinary research can be catalysed from an institutional perspective. Here, we expand on their messages by critically reflecting on our experience in this research to provide insights on how to operationalize interdisciplinary work in practice.

#### (i) A shared impact agenda

The research team was brought together through the long-term and well-structured programme of the CRCWSC, which has an explicit agenda for transforming urban water management to support water sensitive cities. This provided a valuable starting point for attracting researchers that are passionate about driving real-world change and helping our team stay connected and inspired through the challenges of working closely with other disciplines and industry stakeholders. This impact agenda positioned water at the centre of each discipline's research process, helping us keep focus on the range of water sensitive city outcomes that were important for local stakeholders when developing and considering flood resilience solutions, where our individual disciplinary perspectives were likely to optimize one particular objective over others. For example, the modelling research in isolation would have focused on analysis of flood damage costs, compared with the social science and urban design orientation towards broader amenity, social welfare and ecological health benefits. A shared impact agenda also helped us synthesize and communicate our research findings in ways that were relevant and compelling for local stakeholders. This has maximized the potential for enduring impact of the research by engendering a sense of ownership for the participating community, municipalities and other agencies.

#### (ii) A place-based approach

A focus on Elwood and the Elster Creek catchment facilitated integrated thinking among the research team within a particular spatial context, through which interwoven social, technical and environmental challenges could be understood and solutions developed. Our place-based approach was underpinned by processes informed by systems thinking and design thinking, helping us to think holistically and creatively to come up with innovative solutions. Our place-based approach helped us navigate and integrate the different spatial and temporal scales that each research activity needed to engage with. For example, the modelling considered the whole Elster Creek catchment over several decades, the community visioning considered the local suburb of Elwood over several decades, and the urban design exploration considered both local and catchment scales but with a focus on shorter-term implementation opportunities. Each perspective helped create the full suite of solutions that would reduce flood risk while delivering on broader community aspirations through both incremental and systemic change.

#### (iii) A common conceptual framework

Early in the collaboration, we developed a conceptual framework that explicated the shared outcomes, inputs, outputs and information flows that would underpin our interdisciplinary research. This was critical for clarifying how we would take advantage of the synergies and differences between disciplines and how they could lead to integrated solutions. In practice, this involved articulating how each team would work with different data, the insights that would be produced and how they would be used. This required us to establish a common understanding of key terminology used to discuss our methods and outputs; for example, even fundamental terms to individual disciplines such as ‘design’ and ‘model’ were initially understood differently by different parts of the research team. The conceptual framework enabled us to identify and implement periodic cycles of mono-disciplinary research followed by multi-discipline engagements, and then ultimately significant combinations of genuine interdisciplinarity. These iterations enabled understanding and effectiveness between disciplines to grow over time and allowed us to develop both depth and breadth of knowledge required for integration. The different research timelines of each discipline required management within this process, so that key inputs across disciplines could be provided in a timely manner. This operational question needed consideration from an early stage to ensure individual activities were organized to deliver efficiently on the integrated outcomes, and the timing and sequencing of information flows did not always work out as planned, so the team needed to be adaptive as the process unfolded.

#### (iv) A constructive team culture

We quickly realized that our interdisciplinary research would have high transaction costs, requiring significant periods of engagement between teams to establish common ground, agree on focus and direction, and ensure the benefits of collaboration would outweigh the costs. We focused on using plain language in communicating with each other, avoiding technical jargon that would obfuscate meaning. Success required us to genuinely respect the processes and contributions of each other's disciplines and to avoid creating implicit hierarchies between perspectives. This was not always easy and needed each individual researcher to adopt a receptive mindset, commit to open dialogue and be driven by a generosity of spirit through the collaboration.

## 6. Conclusion

This paper has shown that successful adaptation to ensure a city's flood resilience and long-term liveability will involve a range of technical, urban design, policy and social measures to retreat from, accommodate and protect against flooding. The CRCWSC research platform has enabled a novel integrated and interdisciplinary approach for identifying and developing a diverse suite of these measures for Elwood, involving processes that (i) engaged with the community to understand their concerns, aspirations and priorities, (ii) used modelling techniques to test the performance, robustness and economic viability of proposed adaptation solutions, (iii) designed specific measures to densify and utilize urban forms in ways that implement the proposed solutions while responding to the local contextual conditions and (iv) reflected on the governance arrangements and principles needed to improve flood resilience in the Elster Creek catchment.

In addition to scholarly insight on the value of interdisciplinary perspectives for improving flood resilience, the research has led to a promising new flood risk governance arrangement for the Elster Creek catchment, advancement of which will require stakeholders to maintain commitment to, and eventually embed institutionally. It has also arguably empowered the participating group of local Elwood residents, through increasing their water literacy and building their capacity to participate in a more sophisticated engagement with the local government and relevant state agencies on flood resilience issues. This development was both welcomed and greeted with caution by agencies, given its challenge to traditional power dynamics between community and government, as well as the need for careful communication about the potential costs and feasibility of adaptation measures preferred by the community participants. These impacts, which reflect a strengthened local knowledge base for increasing infrastructural resilience as well as enhanced social resilience of the community, highlight the potential of interdisciplinary research to drive positive change in the real world, as well as implementation risks that need to be managed upon the adoption of innovative approaches.

While the value of interdisciplinary approaches is being increasingly recognized in urban drainage and flood management scholarship and practice (e.g. [77,78]), there are few published examples of scientifically robust socio-technical processes and analyses to inform management strategies (see [79] for a recent exception). The approach taken in this paper therefore provides a preliminary model for developing flood resilience strategies in other cities. Our approach aims to harness the benefits of involving the community as partners in flood risk governance, develop adaptation measures based on quantified and detailed flood risk assessments and performance testing, design adaptation solutions that fit within the specific urban forms and local socio-technical context, and tailor governance structures and processes to deliver liveability and resilience outcomes. Our experience, however, highlights the importance of carefully navigating the challenges that come with interdisciplinary work, and we recommend that integrated research teams give particular attention to defining their impact agenda, developing a place-based focus, developing a common conceptual framework to guide the research, and fostering a constructive team culture. We note, however, that this interdisciplinary process takes time and resources, which may not be possible or necessary in every case.

There are a number of potentially fruitful avenues for further research. Our research method did not provide community with the opportunity to incorporate results from the modelling analyses and design processes into their envisioning discussions. (However, the integrated results were displayed in a public exhibition, so participants were able to see the findings.) Further process innovation is therefore warranted to explore the potential of incorporating iteration between the different disciplinary-led activities, for example through presentation of pre-prepared results, as well as through the real-time use of simplified models to assist in the visualization of adaptation measures under different scenarios. It is also worth exploring the potential of bringing together the community and professionals from key agencies with water responsibilities, so they can engage with and learn from each other. These types of integrated activities would have significant value in breaking down barriers between community members, engineering and design professionals and decision-makers to enable thorough exploration of the full suite of adaptation strategies for improving flood resilience in local areas and to establish long-term partnerships. Such an approach would rely on well-designed and sensitively facilitated processes, and tools that allow for clear communication and transparent assessment of potential adaptation measures. Adaptation pathways analysis is a promising example of such tool, able to contribute to garnering support for flexible, long-term plans and keeping options open under uncertainty. Yet, there remain a number of implementation challenges to be addressed in the tool's operationalization, including the timely detection of tipping points and the inclusion of measures that can help smoothen transitions when there is a change in strategic direction or stakeholder commitment [80]. Similarly, while our study scope focused on analysis of flood damage costs, valuing the broader liveability benefits of proposed adaptation measures using the modelling results and non-market valuation tools and evidence would provide additional insight to help decision-makers prioritize investments (for example, the CRCWSC's Investment Framework For Economics of Water-Sensitive Cities (INFFEWS) includes the Values Tool, a navigable database of non-market values [81]). A further disciplinary perspective that would be valuable to support this innovation is information technology, for example, through developing three-dimensional visualizations and gaming approaches to explore adaptation measures under different future scenarios to further support interagency communication, public engagement and options analysis.

To conclude, this paper has demonstrated the value and importance of an interdisciplinary approach to address flood risk in cities, particularly when adaptation responses must typically also deliver other benefits that reflect the community's broader aspirations for a city's liveability and sustainability. There is a clear need for further research on the development and use of tools and processes to enable the development of integrated adaptation strategies for flood resilience and other urban water management challenges.

## Data accessibility

This article has no additional data.
